# Ophthalmic Bimatoprost-Loaded Niosomal In Situ Gel: Preparation, Optimization, and In Vivo Pharmacodynamics Study

**DOI:** 10.3390/polym15214336

**Published:** 2023-11-06

**Authors:** Mohammed F. Aldawsari, Ehssan H. Moglad, Hadil Faris Alotaibi, Hamad M. Alkahtani, El-Sayed Khafagy

**Affiliations:** 1 Department of Pharmaceutics, College of Pharmacy, Prince Sattam Bin Abdulaziz University, Al-Kharj 11942, Saudi Arabiae.moglad@psau.edu.sa (E.H.M.); 2 Department of Microbiology and Parasitology, Medicinal and Aromatic Plants Research Institute, National Center for Research, Khartoum 2404, Sudan; 3 Department of Pharmaceutical Sciences, College of Pharmacy, Princess Nourah Bint AbdulRahman University, Riyadh 11671, Saudi Arabia; hfalotaibi@pnu.edu.sa; 4 Department of Pharmaceutical Chemistry, College of Pharmacy, King Saud University, Riyadh 11451, Saudi Arabia; ahamad@ksu.edu.sa; 5 Department of Pharmaceutics and Industrial Pharmacy, Faculty of Pharmacy, Suez Canal University, Ismailia 41522, Egypt

**Keywords:** bimatoprost, in situ gel, glaucoma, niosomes, ocular delivery

## Abstract

This study aimed at formulating the antiglaucoma agent, Bimatoprost (BMT), into niosomal in situ gel (BMT-ISG) for ocular delivery. Niosomes containing cholesterol/span 60 entrapping BMT were fabricated using a thin-film hydration method. The fabricated niosomes were optimized and characterized for entrapment efficiency (%EE) and size. The optimized BMT-loaded niosomal formulation prepared at a cholesterol/span 60 ratio of 1:2 exhibited the highest entrapment (81.2 ± 1.2%) and a small particle size (167.3 ± 9.1 nm), and they were selected for incorporation into in situ gelling systems (BMT-ISGs) based on Pluronic F127/Pluronic F68. Finally, the in vivo efficiency of the BMT-ISG formulation, in terms of lowering the intraocular pressure (IOP) in normotensive male albino rabbits following ocular administration, was assessed and compared to that of BMT ophthalmic solution. All the formulated BMT-ISGs showed sol–gel transition temperatures ranging from 28.1 °C to 40.5 ± 1.6 °C. In addition, the BMT-ISG formulation sustained in vitro BMT release for up to 24 h. Interestingly, in vivo experiments depicted that topical ocular administration of optimized BMT-ISG formulation elicited a significant decline in IOP, with maximum mean decreases in IOP of 9.7 ± 0.6 mm Hg, compared to BMT aqueous solution (5.8 ± 0.6 mm Hg). Most importantly, no signs of irritation to the rabbit’s eye were observed following topical ocular administration of the optimized BMT-ISG formulation. Collectively, our results suggested that niosomal in situ gels might be a feasible delivery vehicle for topical ocular administration of anti-glaucoma agents, particularly those with poor ocular bioavailability.

## 1. Introduction

Efficient ocular drug delivery is a great challenge for ophthalmologists and drug-delivery scientists. Poor drug bioavailability is a major concern associated with ocular dosage forms following topical application. Only a limited fraction of the topically applied dosage may be absorbed due to a number of ocular physiological and anatomical obstacles [1]. In addition, pre-corneal factors such as fluid drainage, lacrimation, and tear dilution can limit the ocular absorption by decreasing the contact time of instilled drugs at the site of action [2]. Furthermore, corneal drug permeability is greatly influenced by drug physicochemical characteristics, such as solubility, lipophilicity, charge, and molecular size [3]. Consequently, in order to overcome the challenges encountered with conventional topically applied ophthalmic formulations, novel ocular drug delivery systems such as liposomes, microneedles, nanomicelles, nanoparticles, nanowafers, and ocular inserts have been adopted to enhance per-corneal residence and thereby enhance the bioavailability of the therapeutic agents [4,5,6].

Bimatoprost (BMT) is a prostaglandin analogue used to treat glaucoma by lowering the increased intraocular pressure (IOP) [3]. In 2001, it had been approved by the FDA for treating ocular hypertension. BMT shows high corneal absorption; however, conventional BMT dosage forms, such as eye drops, have drawbacks such as short precorneal residence duration and thereby poor bioavailability. Niosomes are non-ionic surfactant-based bilayered vesicles that could encapsulate both hydrophilic and lipophilic drugs. Drug delivery to the ophthalmic sites through a noisome has recently attracted immense interest since it primarily enhances ocular bioavailability, lowers systemic adverse effects, and grants localized action due to its tiny size and less penetrability [7,8,9]. When compared to liposomes, niosomes have several benefits for ocular drug administration such as higher chemical/physical stability, a lack of problems associated with sterilization, and large-scale production encountered with liposomes [7]. Furthermore, niosomes are non-immunogenic, improve drug efficacy through prolonged action and targeted administration [10], have a flexible shape, and are biocompatible and biodegradable in nature [11].

In situ gelling systems are polymer-based viscous liquids that change from a sol to gel state upon application to the human body, owing to changes in physicochemical parameters such as temperature, ionic strength, or pH [12]. Recently, they have gained increased attention as an attractive class of responsive drug delivery systems for various biomedical and pharmaceutical applications, particularly for topical ocular delivery [13,14]. Basically, in situ ocular gels exist in the liquid form before installation into the eye; however, they transferred into a visco-elastic gel once applied into the eye, promoting sustained drug release. They offer the advantages of being easily administered, reducing dosing frequency, and increasing patient compliance [13]. Furthermore, compared to conventional topical ocular formulations, in situ ocular gels promote efficient pre-ocular retention and significantly reduce drug drainage through the naso-lacrimal duct, and thereby, reduce the possibility of eliciting systemic side effects [14,15]. Most importantly, the biomedical application of in situ gels can further be amended by incorporating drug nanoparticles into in situ gelling systems with the aims of prolonging drug release and improving therapeutic outcomes of patients [16,17].

Pluronics^®^ are synthetic block copolymers consisting of hydrophobic poly (propylene oxide) and hydrophilic poly (ethylene oxide) arranged in a triblock structure. These polymers are amphiphilic in nature, having surface active characteristics, and they have the ability to interact with biological membranes [18]. Pluronics^®^ may self-assemble in aqueous solutions to create micelles, which have found uses in the solubilization of poorly soluble drugs [19,20]. Interestingly, Pluronics^®^ are also known to create gels in situ in response to temperature increases [21,22]. Because Pluronics^®^ are translucent, and do not interfere with normal vision, they are best suited for ophthalmology applications. Several ocular formulations, including Pluronic F127 in combination with other copolymers such as cellulose derivatives [23], chitosan [24] or alginate [25], have been described.

The objective of this study was to develop novel BMT-loaded niosomes and incorporate them into an in situ gel for ocular drug delivery, in order to enhance the ocular bioavailability of BM. The thermosensitive polymer Pluronic^®^ F127 (PF127), in combination with the co-polymer Pluronic^®^ F68 (PF68), was used for the formulation of the in situ gel system. The in vivo efficiency of the optimized formulation was then examined via in vivo pharmacodynamic study and ocular irritation tests.

## 2. Materials and Methods

### 2.1. Materials

Bimatoprost (BMT), cholesterol, Pluronic^®^ F127 (PF127), Pluronic^®^ F68 (F68), and Span 60 were purchased from Sigma-Aldrich (St. Louis, MO, USA). Other reagents and chemicals were of analytical grade.

### 2.2. Preparation of BMT-Loaded Niosomes

Thin-film hydration method was implemented for the fabrication of Bimatoprost (BMT)-loaded niosomes [26]. Briefly, definite weights of cholesterol and Span 60, at different ratios (1:1, 1:1.5, and 1:2), were dissolved in chloroform/methanol mixture. The organic solvent mixture was then evaporated under vacuum at 60 °C leaving a dry thin film. The thin film was hydrated with 20 mL of phosphate-buffered saline (PBS) pH 7.4 containing the drug. Eventually, the niosomal dispersion was subjected to sonication (5 cycles of 2 min) at 60 °C to obtain BMT-loaded vesicles.

### 2.3. Experimental Design

A central composite design (CCD) was implemented to optimize BMT-loaded niosomes using Design-Expert^®^ software (version 12, StatEase Inc., Minneapolis, MN, USA) and to explore the influence of independent formulation variables, namely, drug concentration (X_1_) and cholesterol:Span 60 ratio (X_2_), on the specified formulation characteristics, namely, vesicle size (Y_1_) and entrapment efficiency percentage (Y_2_). The impact of each independent variable on product characteristics was tested at 3 levels (Table 1). The design comprises 13 runs prepared with different drug concentrations and cholesterol:Span 60 ratios (Table 2).

### 2.4. Characterization of BMT-Loaded Niosomes

#### 2.4.1. Vesicle Size, Polydispersity Index (PDI), and Zeta Potential Determination

Vesicle size, PDI, and surface charge were determined at room temperature by Malvern Zetasizer (Nano-ZS 90, Worcestershire, UK). Test samples were diluted 1:100 *v*/*v* with deionized water prior to measurements [27].

#### 2.4.2. Drug Entrapment Efficiency (EE%)

Ultracentrifugation technique was employed to determine the amount of BMT entrapped within niosomal vesicles. Briefly, niosomal dispersion was centrifuged at 15,700× *g* rpm for 45 min at 4 °C to separate free drug in the supernatant from entrapped drug. The concentration of free drug in the supernatant was analyzed using Ultraviolet–visible (UV–Vis) spectrophotometer (Shimadzu, Kyoto, Japan) at λ_max_ 294 nm [28] using a preconstructed calibration curve established by testing known serial concentrations of BMT (Figure S1). Equation (1) was used to calculate the entrapment efficiency (%) [29]:

(1) $$\%\;\mathrm{Entrapment}\;\mathrm{efficiency}=\frac{\mathrm{Amount}\;\mathrm{of}\;\mathrm{entrapped}\;\mathrm{drug}}{\mathrm{Total}\;\mathrm{initial}\;\mathrm{amount}\;f\;\mathrm{drug}}\times 100$$

The percentage drug loading of the formulations was also calculated using Equation (2):

(2) $$\%\;\mathrm{Drug}\;\mathrm{loading}=\frac{\mathrm{Entrapped}\;\mathrm{drug}\;\mathrm{weight}}{\mathrm{Total}\;\mathrm{niosome}\;\mathrm{weight}}\times 100$$

#### 2.4.3. Differential Scanning Calorimetry (DSC)

A Shimadzu differential scanning calorimeter (Tokyo, Japan) was adopted to examine the thermal characteristics of pure BMT, Span 60, Chol, physical mixture, and the optimized BMT-loaded niosomal formulation and to assess the possible physical interactions between pure drug and individual formulation components (Span 60, and Chol). A definite weight (2 mg) of each sample was placed in an aluminum pan and heated at a constant rate of 10 °C.min^−1^ under a nitrogen atmosphere [30].

#### 2.4.4. Fourier Transform Infrared (FTIR) Spectroscopy

FTIR spectroscopy was adopted to scrutinize the possible interaction between BMT and niosomal components (Span 60 and cholesterol). Infrared spectra were recorded in the range of 500–4000 cm^−1^ at a resolution of 1 cm^−1^.

### 2.5. In Vitro Drug Release Study

The in vitro release pattern of BMT from BMT-loaded niosomes was investigated using a cellulose dialysis tubing (MW cut-off 12,000–14,000 Da). The dialysis bag was filled with a specific volume of optimized BMT niosomes (equivalent to 3 mg BMT) and then suspended in 150 mL simulate tear fluid (STF; pH 7.4). The release medium was maintained at 37 ± 0.5 °C and stirred at 100 rpm. At scheduled time intervals (0.5, 1, 2, 4, 8, 12, 18, and 24 h), 3 mL samples were collected and replenished with an equal volume of STF to maintain sink conditions. The concentration of BMT in the collected samples was analyzed spectrophotometrically at 294 nm.

### 2.6. Physical Stability of BMT-Loaded Niosomes

Stability studies of optimized BMT-loaded niosomes was conducted via monitoring changes in vesicle size, zeta potential and/or drug leaking from optimized niosomal vesicles, upon storage for 90 days. Briefly, BMT-loaded niosomes were stored in a refrigerator at 4 °C for 3 months. The alterations in niosomal vesicle size, surface charge, or drug content were monitored during storage period [29].

### 2.7. Incorporation of BMT-Loaded Niosomes into In Situ Gels (BMT-ISGs)

In situ gelling systems based on Pluronic F127 (PF-127)/Pluronic F68Pluronic^®^ (PF-68) containing BMT-loaded niosomes equivalent to 0.3% *w*/*w* of the drug were prepared by the cold method at a total polymer concentration of 20% *w*/*w* [14]. Accurately weighed amounts of PF-127 and Pluronic^®^ F68 (PF-68), as a co-polymer, were mixed together into cold deionized water at 4 °C. The mixture was continuously stirred at 200 rpm for 2–3 h until a clear homogenous solution was obtained. Accurately weighed amount of optimized BMT-loaded niosomes was then added to the prepared hydrogel mixtures and stored overnight in a refrigerator.

### 2.8. Evaluation of the Prepared BMT-Loaded Niosomal into In Situ Gels (BMT-ISGs)

#### 2.8.1. Visual Appearance and pH

The prepared BMT-ISGs were inspected visually for color, clarity, and homogeneity against a black and white background. The pH measurements were carried out using the pH meter (Jenway, Staffordshire, UK). Results are represented as mean values ± SD.

#### 2.8.2. Drug Content Analysis

An accurately weighed amount of BMT-ISGs was dispersed in a specific volume of PBS (30 mL, pH 7.4) with vigorous shaking for 12 h. The dispersion was then filtered and drug amount in the filtrate was analyzed spectrophotometrically at λ_max_ equal to 294 nm. Equation (3) was used to calculate drug content (%):

(3) $$\mathrm{Drug}\;\mathrm{content}\;\left(\%\right)=\frac{\mathrm{Actual}\;\mathrm{amount}\;\mathrm{of}\;\mathrm{BMT}}{\mathrm{Theoretical}\;\mathrm{amount}\;\mathrm{of}\;\mathrm{BMT}}\times 100$$

#### 2.8.3. Sol–Gel Transition Temperature Determination

Sol–gel transition temperature was assessed using an inversion method [31], where one gram of the ISGs was put into a closed test tube and placed on a thermostatic water bath. The experiment started at 25 °C, and the temperature of the water bath was steadily increased by one degree until the solution converted into the gel. Sol–gel transition temperature is determined when no movement into the liquid was observed upon tilting up the closed test tube at 90 °C.

#### 2.8.4. Determination of the Viscosity of BMT-ISGs

The viscosity of different BMT-ISGs was determined using Brookfield viscometer (Model DV-III, spindle 16, Chandler, AZ, USA). Definite weight samples of different formulations were inserted into a small holder and a perpendicular spindle was revolved at a constant speed of 100 rpm. Viscosity measurements were conducted at 25 °C and 37 °C.

### 2.9. In Vitro Release of BMT from Different BMT Formulations

The in vitro release profiles of BMT from BMT solution, BMT-loaded niosomes, or selected BMT-ISG formulation were assessed using the USP dissolution test apparatus-I with certain modifications. Briefly, accurately weighed amounts of various formulations (equivalent to 3 mg of BMT) were placed in glass cylinders covered with a cellulose membrane, pre-soaked in PBS (pH 7.4), from one end and fixed to the shaft of the dissolution apparatus from the other end. The glass cylinders were then immersed in beakers containing 150 mL of STF (pH 7.4) as a dissolution medium. The cylinders were adjusted to rotate at 100 rpm at 37 ± 1 °C. The 3 mL aliquots were collected at scheduled time intervals and replenished with fresh medium to maintain sink conditions. The concentration of BMT in each sample was analyzed spectrophotometrically at 294 nm.

### 2.10. Ex Vivo Corneal Permeability Study

Ex vivo corneal permeation studies of either optimized BMT-loaded niosomes or BMT-ISG were conducted using freshly excised goat cornea and compared with that of BMT solution. Briefly, fresh goat eyeballs were procured from a slaughterhouse and kept in normal saline at 4 °C. The cornea was gently removed using forceps and scissors and fixed in between the donor and receptor compartments of a Franz diffusion cell. Accurately weighed amounts of various formulations (equivalent to 3 mg of BMT) were placed on the corneal membranes in the donor chamber while the receptor chamber was filled with freshly prepared STF (pH 7.4) maintained at 35 ± 1 °C and under magnetic stirring of 100 rpm. Aliquot samples (1 mL) were collected at predetermined time points and instantly replaced with an equal volume of STF. The concentration of BMT in each sample was analyzed at 294 nm.

### 2.11. In Vivo Studies

#### 2.11.1. Animals

Male albino rabbits (2–2.5 kg) were used in in vivo experiments. The animals were housed under specific environmental conditions (a 12 h on/off light schedule, 25 ± 0.5 °C, and 65% relative humidity). Rabbits were fed a routine rabbit diet with free access to water. The study protocol was approved by Ethical Committee, Prince Sattam Bin Abdulaziz University, Al-Kharj, KSA (approval number: 048/2022).

#### 2.11.2. In Vivo Pharmacodynamic Study

The in vivo pharmacodynamic efficacy of different BMT formulations, in terms of lowering intraocular pressure, was assessed using male albino rabbits. Briefly, animals were categorized into three groups (n = 6). Group I was ocularly treated with BMT ocular solution. Group II was treated with optimized BMT-loaded niosomes, while Group III was treated with BMT-ISG. All animals were treated with 50 μL of 0.03% *w*/*v* of various BMT formulations. All formulations were applied in the left eye of each animal, while the right eye was kept untreated (control). Schiotz Tonometer (Rudolf Riester GmbH, Jungingen, Germany) was used to measure the IOP before administration and at scheduled time intervals (0, 0.5, 1, 2, 3, 4, 5, and 6 h) post formulation installation. The mean change in IOP (∆%) between the two eyes of each animal was calculated using the following equation [32]:

$$\mathrm{The}\;\mathrm{mean}\;\mathrm{change}\;\mathrm{in}\;\mathrm{IOP}\;(∆\;\mathrm{IOP})={\mathrm{IOP}}_{\mathrm{control}\;\mathrm{eye}}-{\;\mathrm{IOP}}_{\mathrm{dosed}\;\mathrm{eye}}$$

In addition, key pharmacodynamic parameters such as the mean maximal effect (E_max_), time to E_max_ (T_Emax_), and area under the effect curve (AUC_E 0–8h_) were compared between treatment groups.

#### 2.11.3. Ocular Irritation Test

Ocular irritation following treatment with different BMT formulations was investigated using the modified Draize test [33]. Briefly, a total of six male albino rabbits were categorized into two groups, three animals in each. The two groups were treated with BMT solution and BMT-ISG. At specific time points post formulation application, each rabbit was observed for signs of irritation (swelling discharge, redness, iris and corneal lesion, and conjunctival chemosis). The following scores were applied to assess the irritation severity: Score 0 indicates no redness, inflammation, or excessive tearing; 1 indicates mild redness with inflammation and minimal tears; 2 indicates moderate redness with considerable inflammation and significant tearing; and 3 indicates severe redness with severe inflammation and extensive tearing.

### 2.12. Statistical Analysis

All the experiments were carried out in triplicate and the results obtained are reported as mean ± standard deviation. Statistical significance was determined using Graph-pad prism 7 version with students *t*-test and one way analysis of variance (ANOVA), where *p* < 0.05 was considered to be statistically significant.

## 3. Results and Discussion

### 3.1. Preparation of BMT-Loaded Niosomes

The thin-film hydration method was employed for the fabrication of BMT-loaded niosomes. Preliminary trials were performed to select the appropriate non-ionic surfactant for the formulation of BMT-loaded niosomes. Several surfactants, including Brij 35, Spans, and Tweens, were investigated for their ability to form a physically stable vesicular structure. Our preliminary results revealed that surfactants, such as Tween 60, 80, and Brij 35, that have HLB values ranging from 15 to 17 failed to create a homogeneous thin film, and following hydration, the formed vesicles were unstable, as evidenced by vesicle sedimentation and fast phase separation. On the contrary, niosomes made with span 60 and span 80 were spherical in shape, homogenous in size, and show no phase separation. The high transition temperature and optimal HLB values of Spans (4.3 and 4.7, respectively) might account for their efficient vesicle formation ability [34]. However, niosomes produced with span 80 exhibited considerable aggregation during short-term storage. Consequently, niosomes prepared with Span 60 were used for further investigations.

### 3.2. Experimental Design and Optimization

A central composite experimental design was implemented for the development of BMT-loaded niosomes and the assessment of the influence of two formulation parameters (drug concentration and cholesterol:surfactant (Chol:SAA) ratio) on formulation characteristics. The effect of these two variables on vesicle size and percentage entrapment efficiency (EE%) was examined using 3D surface plots. A total of 13 runs with varying amounts of independent formulation parameters were developed. The vesicle size and EE% of all produced formulations were then analyzed. The optimization goal was set to obtain a formulation with a minimum vesicle size while having the maximum entrapment efficiency percentage (Table 1). Table 2 depicts the attributes of various BMT-loaded niosomal formulations generated using the central composite design.

#### 3.2.1. Impact on Particle Size of Formulation Variables

Generally, niosomes within a size range of less than 200 nm are considered ideal for ocular administration owing to their supreme penetration power through corneal barriers [28]. Herein, all BMT-loaded niosomes showed vesicle sizes ranging from 165.9 ± 6.7 to 219.1 ± 9.6 nm (Table 2). Several polynomial models were used to fit the data for vesicle size (Y1). Statistical analysis (Table S1) depicts that the obtained data were best fitted in the quadratic model. The obtained equation in terms of coded values was as follows:

Vesicle size (Y_1_) = 189.8 + 8.85 X_1_ − 16.47 X_2_ − 0.25 X_1_X_2_ + 0.475 X_1_^2^ + 1.68 X_2_^2^

**Figure 1:**
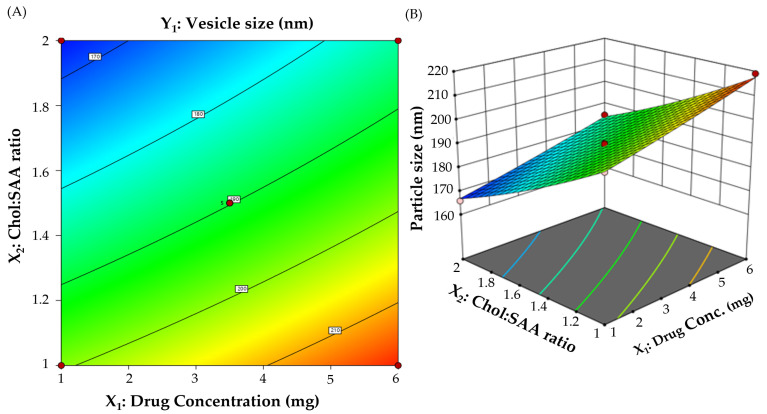
(**A**) Contour and (**B**) 3D plots for the impact of different formulation variables on niosomal vesicle size.

This equation discloses that the drug concentration exerts a synergistic effect on niosomal vesicle size whilst the Chol:SAA ratio exerts an antagonistic effect on the vesicle size. In addition, Figure 1 represents contour and 3D plots that illustrate the influence of formulation variables on the vesicle size of the prepared BMT-loaded niosomes. It is evident that, at a fixed Chol:SAA ratio, the sizes of the niosomes remarkably increased upon increasing drug concentration. The vesicle size of F1 (219.1 ± 9.6 nm), prepared at a drug content of 6 mg, was considerably higher than that of niosomes prepared at a drug content of 1 mg (F5; 199.1 ± 7.8 nm). The increase in drug amount being entrapped within the niosomal vesicle might ascribe the increase in the vesicle size [35]. Similar results were obtained by Mohanty et al. who verified a significant increase in the size of naproxen-loaded niosomes upon raising the drug concentration [36].

Regarding the influence of the Chol:SAA ratio on vesicle size (Y_1_), it was clear that elevating the Chol:SAA ratio from 1:1 to 1:2 resulted in a pronounced negative effect on the vesicle size. At a fixed drug concentration, the vesicle size of niosomes fabricated at a Chol:SAA ratio of 1:1 (F5; 199.1 ± 7.8 nm) was significantly higher than that of niosomes fabricated at a Chol:SAA ratio of 1:2 (F8; 165.9 ± 6.7 nm). At a Chol:SAA ratio of 1:2, the relatively high concentration of Span 60 would favor the production of micellar structures with smaller sizes rather than niosomal vesicles [37]. These results are in agreement with those stated by Aldawsari et al., who highlighted the negative impact of increasing the Chol:SAA ratio from 1:1 to 1:2 on the vesicle size of cetirizine-loaded niosomes [26].

#### 3.2.2. Impact on Percentage Entrapment Efficiency (EE%) of Formulation Variables

Entrapment efficiency is an important factor in characterizing colloidal systems. Several formulation parameters, including the drug concentration and Chol:SAA ratio, were changed to achieve the best entrapment efficiency. As illustrated in Table 2, EE% ranged from 66.2 ± 1.9% to 84.1 ± 3.2%. The impact on entrapment efficiency (Y_2_) of different formulation variables is represented by the following quadratic polynomial equation:

Entrapment efficiency % (Y_2_) = 77.4 + 3.61 X_1_ + 3.21 X_2_ − 0.275 X_1_X_2_ − 3.56 X_1_^2^ + 1.01 X_2_^2^

**Figure 2:**
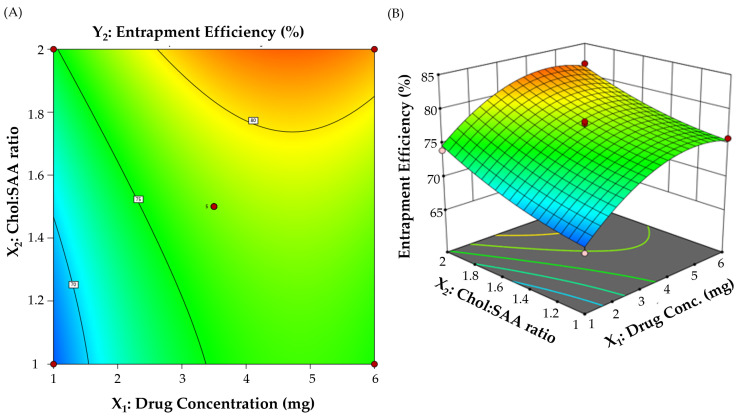
(**A**) Contour and (**B**) 3D plots for the impact of different formulation variables on niosomal entrapment efficiency percentage.

Additionally, the effect of formulation variables on EE% was graphically plotted using the contour and 3D surface plot (Figure 2). It was clear that both the drug concentration and Chol:SAA ratio had a positive impact on BMT entrapment within niosomal vesicles. At a fixed Chol:SAA ratio, there was a dramatic increment in EE% upon raising drug concentration from 1 to 6 mg. The EE% of F5, prepared with 1 mg BMT, was significantly lower than that of F1, prepared with 6 mg BMT (66.9 ± 1.1% vs. 75.8 ± 1.7%). The remarkable increase in the percentage of BMT entrapped within niosomes might be accounted for by the saturation of the hydration medium with BMT, enforcing BMT to be encapsulated within niosomes [38,39].

In the same context, the entrapment efficiency increases with a rise in the Chol:SAA ratio. At a fixed drug concentration, increasing the Chol:SAA ratio from 1:1 to 1:2 triggered a remarkable increase in BMT entrapment within niosomes. The EE% of F5, prepared at a Chol:SAA ratio 1:1, was 66.9 ± 1.1%, which was remarkably lower than that of F8 (EE% 74.0 ± 1.8%), prepared at Chol:SAA 1:2. These findings might be ascribed, at least in part, to the increase in the Span 60 concentration, which would help in enhancing the drug solubilization and thereby boosting drug entrapment within niosomal vesicles. Similar findings were disclosed by Ghazwani et al. who underscored the synergistic effect of the surfactant concentration on the entrapment of carvacrol oil with niosomal vesicles [40].

#### 3.2.3. Numerical Optimization of BMT-Loaded Niosomes

A numerical optimization procedure was carried out using Design-Expert^®^ software to obtain an optimized niosomal formulation that fulfills the required response (Table 1). A drug concentration of 3.6 mg and a Chol:SAA ratio of 1:2, obtained at a desirability of 0.845, were recommended for the preparation of optimized BMT-loaded niosomes. The optimized formulation was found to meet the design constraints of the maximum entrapment efficiency and a minimal particle size. The measured % EE was 81.2 ± 1.2% and the vesicle size was 167.3 ± 9.1 nm, both of which were comparable to the expected values of entrapment efficiency and vesicle size (81.7% and 175.3 nm, respectively).

### 3.3. Characterization of Optimized BMT-Loaded Niosomes

#### 3.3.1. Vesicle Size, Polydispersity Index (PDI), and Zeta Potential

**Figure 3:**
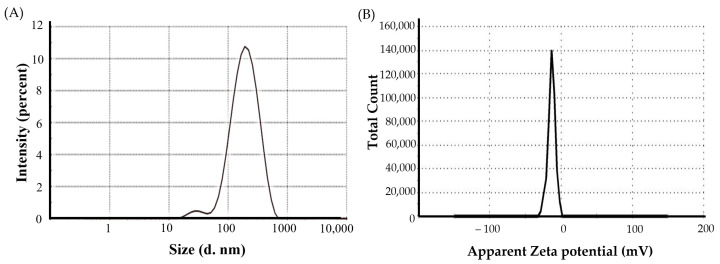
(**A**) vesicle size and (**B**) zeta potential of optimized BMT-loaded niosomes.

The mean vesicle size of optimized BMT-loaded niosomes was 167.3 ± 9.1 nm, which is within the ideal size range (<200 nm) for ocular administration [41]. The vesicle size distribution (PDI) for the optimized BMT-loaded niosomes was 0.269 ± 0.09, signifying homogenous size distribution [42]. The zeta potential was determined to be −12.4 ± 1.9 mV, implying fair colloidal stability. Figure 3A,B illustrate the size and zeta potential of the optimized formulation, respectively.

#### 3.3.2. Entrapment Efficiency and Drug Loading

The efficient entrapment of the drug within a niosomal vesicle is a crucial criterion for characterizing niosomal dispersions. The calculated entrapment efficiency of BMT within an optimized niosomal formulation was 81.2 ± 1.2%, which indicates efficient encapsulation of the drug within niosomes. The drug loading percentage of BMT within niosomes was calculated to be 3.46 ± 0.23%.

#### 3.3.3. DSC Study

**Figure 4:**
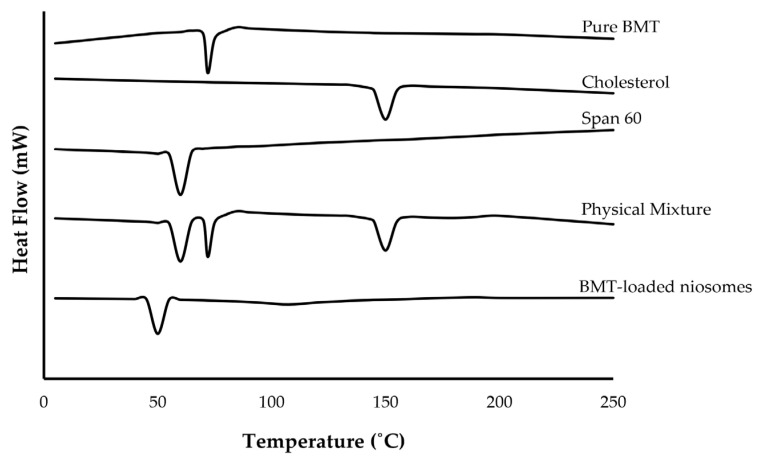
DSC thermograms of different components of optimized BMT-loaded niosomes.

The DSC analysis was performed to explore the thermal properties of pure drug, cholesterol, Span 60, and optimized BMT-loaded niosomes, as well as possible interactions between the components. The results of the DSC analysis of pure BMT showed a characteristic endothermic peak at 71.9 °C, which corresponded to its melting point (Figure 4). Span 60 and cholesterol showed characteristic peaks at 54.6 and 148.2 °C, respectively, corresponding to their melting points. In addition, the physical mixture showed all the characteristic endothermic peaks of pure drug and formulation components (Span 60 and cholesterol), nullifying the existence of any drug–excipient incompatibility between the drug and formulation components. Of note, no characteristic endothermic peaks were identified for BMT in the thermogram of BMT-loaded niosomes, suggesting that BMT was entirely entrapped within niosomal vesicles [26].

#### 3.3.4. FTIR Studies

The FTIR spectra of BMT, cholesterol, Span 60, and optimized BMT-loaded niosomes were investigated for possible interactions between BMT and niosomal components (Figure S2). The BMT spectrum revealed distinctive peaks such as O−H stretching (3410 cm^−1^), N−H stretching (3329 cm^−1^), C−H stretching (3089 cm^−1^), and N-C=O (amide) stretching (1610 cm^−1^) [28]. Characteristic peaks were observed at 1170, 1744, and 2878–2939 cm^−1^ in the spectrum of Span 60, corresponding to −C−COO, C=O and aliphatic C−H, respectively. For cholesterol, the FTIR spectrum showed distinct peaks of C−O stretching (1053 cm^−1^), aliphatic C−H stretching (2800–3000 cm^−1^), and a broad peak of O−H (3380 cm^−1^). Of interest, the spectrum of optimized BMT-loaded niosomes showed most of the characteristic peaks of both BMT and niosomal components, suggesting the absence of any chemical interactions between them.

### 3.4. Stability Studies of Optimized BMT-Loaded Niosomes

The physical stability of BMT-loaded niosomes was investigated in terms of changes in vesicle size, zeta potential, and EE% upon 3 months storage at 4 °C. When compared to freshly prepared niosomes, no significant changes in entrapment efficiency, vesicle size, and zeta potential were found throughout a 1- and 3-month storage period, as shown in Table S2. These results underscored the stability of optimized BMT-loaded niosomes.

### 3.5. Formulation of BMT-Loaded Niosomal In Situ Gel (BMT-ISG)

In this study, the optimized BMT-loaded niosomal formulation was incorporated into an in situ gel formulation to facilitate its application, prolong the residence time onto the corneal surface after application, and subsequently, augmenting drug penetration through the cornea. For such a purpose, in situ gelling systems based on Pluronic^®^ F127 (PF-127) in combination with Pluronic^®^ F68 (PF-68) at various concentrations, ranging from 0.5 to 2.5% *w*/*w*, were employed for the preparation of BMT-loaded niosomal in situ gel (BMT-ISG). In addition, the impact of co-polymer (PF-68) concentration of the formulation attributes of BMT-ISGs was investigated and adopted to obtain an optimized formula of the BMT-ISG. Of note, the concentrations of the co-polymer (PF-68) used were selected based on our preliminary trials. Where, at a co-polymer concentration lower than 0.5% *w*/*w*, the viscosity of the formed in situ gel was very low. On the other hand, increasing the co-polymer concentration to more than 2.5% *w*/*w*, resulted in the formulation being too thick, which adversely hindered the easy application of the formed BMT-ISGs. Accordingly, a co-polymer concentration ranging from 0.5 to 2.5% *w*/*w* was tested for the formulation of BMT-ISGs.

### 3.6. Evaluation of BMT-ISGs

#### 3.6.1. In Situ Gel Visual Examination

The prepared BMT-ISGs were evaluated visually prior to and after loading with a BMT-loaded niosomes for color, clarity, and homogeneity. Before loading, plain ISGs showed a clear, homogenous, and colorless appearance, however, after loading with BMT niosomes, the ISGs became opaque with a white color.

#### 3.6.2. pH

For ophthalmic preparations, it is crucial to ensure that the pH of the fabricated formulation falls within the normal ocular comfort range (pH range of 6.6 to 7.8) to avoid eye irritation and discomfort. In this study, the mean pH values of all prepared BMT-ISGs were within the acceptable range (6.42 ± 0.02 to 7.19 ± 0.03) (Table 3), nullifying the possibility of elicitation of eye irritation/discomfort upon instillation into eye.

#### 3.6.3. Drug Content

All the prepared ISGs showed high drug content ranging from 97.3 ± 0.6% to 99.4 ± 0.3% (Table 3). This indicates that BMT-loaded niosomal vesicles were uniformly distributed within the prepared ISGs, and the method was simple and reproducible.

#### 3.6.4. Sol–Gel Transition Temperature (T_c_) of BMT-ISGs

The sol–gel transition temperature (T*_c_*) of BMT-ISGs formulations is a crucial parameter in predicting the ability of applied formulations to be transferred into gel state at the ocular temperature upon installation. Generally, an ophthalmic in situ gel should have Tc higher than room temperature (25 °C) to be easily instilled into the eye but converted into a gel form at pre-corneal temperature (37 °C). A T*_c_* higher than 37 °C is not preferred to be applicable in the eye, since the applied formulation would remain in the sol state following ophthalmic administration and would suffer from nasolacrimal drainage before exerting its therapeutic effect. Herein, the prepared formulation had T*_c_* ranging from 28.1 ± 0.5 °C to 40.5 ± 1.6 °C (Table 3). The gelling capacity increased with the increasing concentration of the PF-8 co-polymer. Among various in situ gel formulations, in situ gels prepared at a PF-68 concentration of 0.5–1.5% (F2, F3 and F4) are considered optimal for the ophthalmic in situ gel, where the conversion from the sol state to the gel state at ocular temperature would be favored by the gradual desolvation of the polymer, increased micellar aggregation, and higher entanglement of the polymer network with raising the temperature [43].

#### 3.6.5. Viscosity of BMT-ISGs

Generally, ophthalmic in situ gels should have an optimum viscosity to ease their instillation into the eye and a quick sol to gel transition [43]. Viscosity measurements were performed before gelling at 25 °C and after gelling at 37 °C. The mean results of the viscosity of the prepared ISGs before and after gelling are summarized in Table 3. With the exception of F6 and F7, all BMT-ISG formulations exerted low viscosities at room temperature, ranging from 105.9 ± 9.1 to 144.6 ± 9.6 cp. Of interest, a drastic increase in formulation viscosity was observed upon elevating the temperature to 37 °C, where gelling will occur [43]. Furthermore, with a rise in the concentration of the PF-68 co-polymer, there was a mutual increase in the viscosity of the formed BMT-ISGs before and after gelling. Such a rise in viscosity could be explained by the interaction between the co-polymers with the micellar entanglement of PF-127, which might trigger the formation of stronger bonds, resulting in an increase in the formulation viscosity. Based on viscosity measurements and other previous parameters, F4 was selected for further studies since it has good gelation capacity at body temperature, an appropriate viscosity at 25 °C, which would facilitate its ocular application, and its maximum viscosity at 37 °C, which would promote prolonged ocular residence of the formulation post its instillation into eyes.

### 3.7. In Vitro Drug Release from Various BMT Formulation

**Figure 5:**
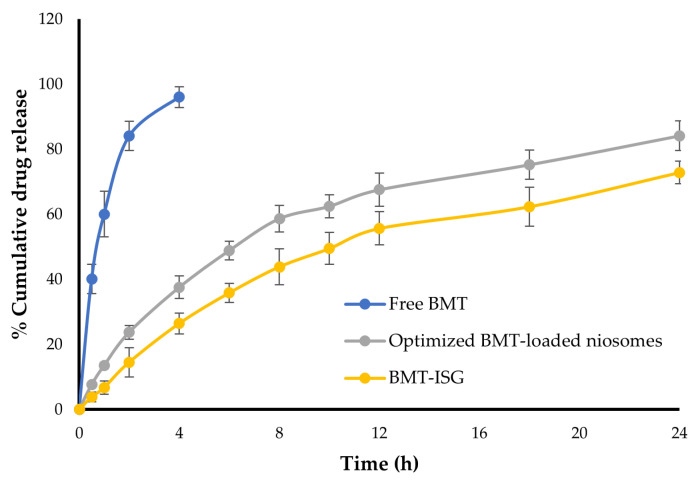
In vitro release of BMT from various BMT formulations.

The in vitro release profiles of optimized BMT-loaded niosomal dispersion, BMT solution, and BMT-ISG formulation (F4) were graphically illustrated in Figure 5. Free BMT showed a rapid release from BMT solution, with ~90% of BMT released at 4 h. By contrary, the encapsulation of BMT within niosomal vesicles significantly delayed BMT release for up to 24 h. BMT-loaded niosomes demonstrated a biphasic release profile, with ~50% of the entrapped BMT released from niosomes during the first 6 h, followed by a sustained drug release over 24 h, with up to 80% of BMT released at 24 h. The initial quick release of BMT might be caused by the rapid release of BMT from the niosome surface, whist the subsequent sustained release phase could be owed to the slow diffusion of drug molecules through niosomal bilayers [40]. Of interest, incorporating BMT-loaded niosomes into ISGs had significantly the sustained drug release, compared to that from parent niosomes. The maximum percentage of BMT released from BMT-ISGs in 24 h was 72.8 ± 3.5%, compared to 84.1 ± 4.6% for BMT-loaded niosomes. This could be related to the gel network and the higher viscosity of the formulation at 37 °C, which might act as a barrier to a rapid drug release leading to a prolonged drug release over 24 h.

The release profiles of BMT from different BMT formulations were analyzed using four kinetic models (Table S3), and the linear regression analysis was employed to obtain the regression coefficients (r^2^) [44]. The calculated r^2^ values remained higher for the Higuchi model, suggesting that the release kinetics of both BMT-loaded niosomal formulation or selected BMT-ISG formulation (F4) were best fitted to Higuchi-diffusion release kinetics. In addition, the Korsmeyer–Peppas model was also used to determine the drug release mechanism from BMT-ISG formulation, and ‘n’ values were found to be >0.43 and <0.85, indicating non-Fickian transport for the selected BMT-ISG formulation, where the release is controlled by a combination of diffusion of the drug and hydration and dissolution of the gel matrix.

### 3.8. Ex Vivo Trans-Corneal Permeation Study

**Figure 6:**
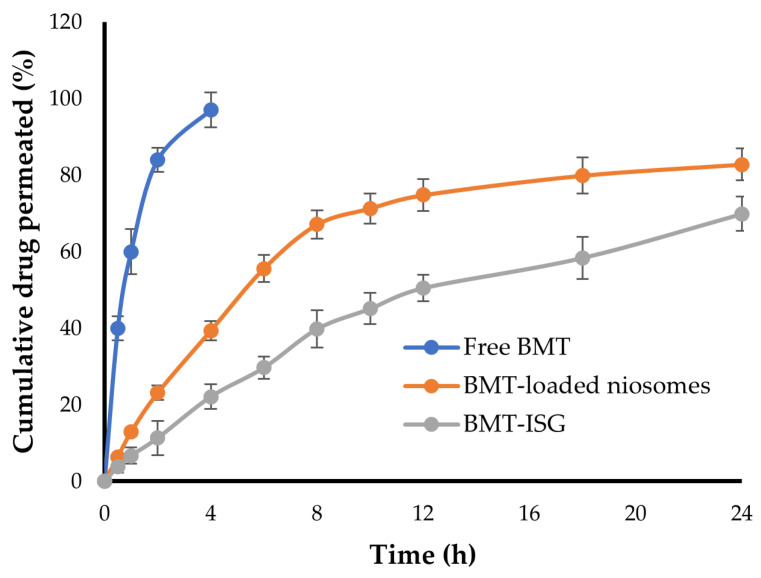
Ex vivo ocular permeability of BMT from various BMT formulations.

Ex vivo drug permeation data of free BMT, niosomal BMT, and BMT-ISGs were obtained using goat corneal membranes. As depicted in Figure 6, free BMT exhibited good ocular permeability; >95% of drugs were permeated at 4 h. Similarly, the niosomal formulation efficiently enhanced BMT permeation through the corneal membrane. The cumulative drug permeated a percentage of 82.8% at 24 h. On the other hand, the maximum cumulative drug release from the BMT-ISG formulation was 69.9% after 24 h, which was lower than that from the niosomal formulation. Nevertheless, the BMT-ISG formulation efficiently sustained drug release for up to 24 h. Such a slow and sustained drug permeation pattern from BMT-ISG formulation could be owed, on the one hand, to the mucoadhesive properties of the ISG formulation that prolong the contact between the formulation and the corneal membrane [45], and on the other hand, to the increased viscosity of the ISG formulation, which could sustain drug release from the formulation. Of note, when compared with in vitro release results, the cumulative amount of BMT penetrating through the corneal membrane from the BMT-ISG formulation was low. This might be ascribed to the lipophilic–hydrophilic barrier imparted by the corneal membrane, which might hinder drug permeation compared to the mechanical barriers imparted by the dialysis membrane [46].

### 3.9. In Vitro Stability Study

The stability of the selected BMT-ISG formulation (F4) was assessed following storage at 4 °C for 8 weeks. Visual appearance, drug content, and pH were investigated. As summarized in Table 4, storage for up to 8 weeks at 4 °C resulted in little difference in visual appearance, pH, and drug content, inferring that the formulation is stable (Table 4).

### 3.10. In Vivo Pharmacodynamic Study

**Figure 7:**
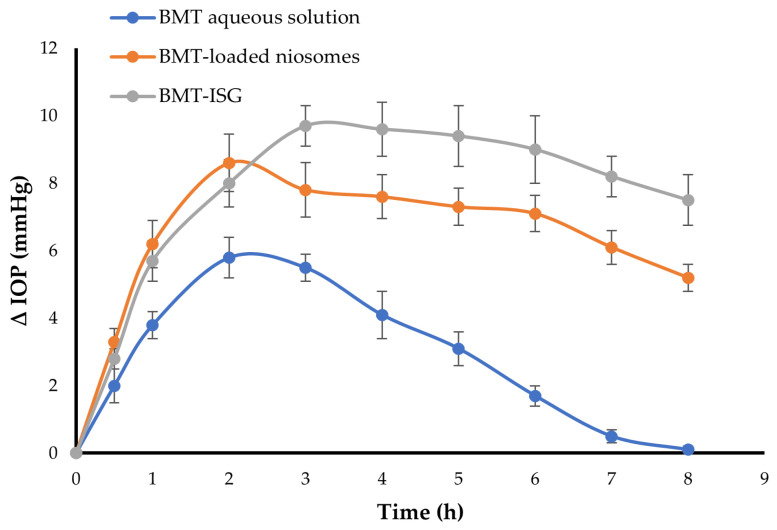
The mean change in IOP induced by treatment with different BMT formulations. Data represent mean ± SD of three independent experiments (n = 6).

In vivo pharmacodynamic study was carried out to scrutinize the in vivo efficacy of the optimized BMT-ISG formula, compared to either BMT-loaded niosomes or BMT aqueous solution, on decreasing IOP. Figure 7 represents the mean change in IOP from the baseline vs. time following treatment with the optimized BMT-ISG formula, BMT-loaded niosomes, or BMT aqueous solution, respectively. As depicted in Figure 7, BMT aqueous solution induced a rapid decrease in the IOP after one hour post ocular instillation and continued for 2 h post treatment, then IOP increased gradually to its initial value after 8 h post-instillation. This complete depletion of the drug effect might be ascribed to washing out/nasolacrimal drainage of the instilled aqueous solution. For the optimized BMT-loaded niosomes, a maximum IOP lowering effect was also observed at 2 h post niosomal dispersion instillation (8.6 ± 0.9 mm Hg), which was superior to the effect of the BMT aqueous solution on IOP. However, such a potent IOP lowering effect was attenuated gradually with time, presumably due to the ocular clearance of niosomal dispersion. Of interest, the BMT-ISG formula elicited a remarkable decline in the IOP values after 3 h with a maximum change of 9.7 ± 0.6 mm Hg, compared to either the BMT aqueous solution (5.5 ± 0.4 mm Hg) or BMT-loaded niosomes (7.8 ± 0.8 mHg). Furthermore, the impact of the decrease in IOP continued until 8 h post-treatment. The average reduction in IOP values for BMT-ISG after 8 h was 7.5 ± 0.8 mm Hg. This means that the inclusion of BMT niosomes into the ISG formulation would sustain drug release for a more prolonged time than either the BMT aqueous form of BMT niosomal dispersion, and subsequently, the instilled dose could be decreased. It is worth noting that during the course of treatment with the BMT-ISG formula, there were no changes in the IOP readings of the untreated eyes, which implies that the impact of the test formula was caused by a local action rather than systemic absorption of the drug.

Key pharmacodynamic parameters of the tested formulations are tabulated in Table 5. As illustrated in Table 5, the mean maximal effects (E_max_) for the BMT aqueous solution, BMT niosomal dispersion, and BMT-ISG formulation were 5.8 ± 0.6 mm Hg, 8.6 ± 0.9 mm Hg and 9.7 ± 0.6 mm Hg, respectively, as recorded at t_Emax_ of 2 h, 2h, and 3 h, respectively. Most importantly, the optimized BMT-ISG formulation showed a higher AUCE_0-8h_ value (63.32 ± 5.8 mm Hg.h) than both the aqueous BMT solution (24.6 ± 3.7 mm Hg.h) and BMT niosomal dispersion (53.272 ± 2.9 mm Hg.h). Of note, although BMT has good corneal absorption [28], the lower ocular bioavailability of BMT aqueous solution might be attributed to its short residence time at the corneal surface (MRT 3.16 ± 0.2 h). The BMT aqueous solution can be easily washed out by tears fluid and may be drained out/in the lacrimal duct, leading to a lower AUC_E0-8h_ value. On the contrary, the comparatively higher AUC_E0-8h_ of BMT niosomal dispersion might be ascribed to the entrapment of BMT within niosomal vesicles, which would enhance the corneal permeability of BMT, compared to the pure drug [11]. Most importantly, the highest AUC_E0-8h_ value observed with the BMT-ISG formulation might be attributed to the enhanced permeability power of niosomes through the corneal surface, along with the prolonged residence time of the BMT-ISG formulation onto the corneal surface (MRT = 12.62 ± 1.3) by virtue of its good muco-adhesion characteristics. Collectively, this significantly higher AUC_E_ of the BMT-ISG formulation would contribute to the superior therapeutic potential of the formulation, compared to the BMT aqueous solution. Similar findings were stated by Obiedallah et al. who demonstrated that incorporating the carbonic anhydrase inhibitor, acetazolamide, within a microsponge-based in situ gel significantly augmented the therapeutic efficacy of acetazolamide in reducing the IOP, compared to the free drug in the gel, following topical ocular administration [14].

### 3.11. Ocular Irritation Test

The Draize rabbit eye test is a commonly used test to scrutinize the probable acute toxicity of chemicals, compounds, and formulations to the eye upon ocular administration. Herein, ocular irritation was investigated following topical ocular administration of the BMT aqueous solution and BMT-ISG formulations. Throughout the test, no signs of ocular irritation such as tearing, redness, or swelling were detected in the three treated groups. These results nullified the irritant potential of test formulations.

## 4. Conclusions

In this study, we attempted to boost the therapeutic potential of bimatoprost (BMT) via its incorporation into the niosomal in situ gel system (BMT-ISG). A BMT-loaded niosomal dispersion was prepared and optimized using a central composite design. The optimized BMT-loaded niosomes were then incorporated into in situ gelling systems based on Pluronic^®^ F127 (PF-127) in combination with Pluronic^®^ F68 (PF-68). The prepared BMT-ISGs showed appropriate sol–gel transition temperatures, facilitating their conversion into gel form at body temperature. In addition, they had acceptable viscosities that enable easy formulation application and prolong the ocular residence time post installation. Furthermore, the prepared BMT-ISGs efficiently sustained drug release compared to their parent free drug. In vivo pharmacodynamic studies verified the potential of the BMT-ISG to lower IOP and to enhance BMT corneal permeability, compared to the BMT aqueous solution. Most importantly, the optimized BMT-ISG formulation was well tolerated by eye tissues as demonstrated by the absence of any signs of irritation post instillation. To sum up, niosomal in situ gel formulations might be a viable delivery vehicle for topical ocular administration of anti-glaucoma agents, particularly those with poor ocular bioavailability.

## Figures and Tables

**Table 1:** Central composite design (CDD) for BMT-loaded niosomes.

Independent Variables	Levels		
	Low (−1)	Medium (0)	High (+1)
X_1_: BMT concentration (mg)	1	3	6
X_2_: Chol:SAA ratio	1:1	1:1.5	1:2
Responses	Constrains		
Y_1_: Vesicle size (nm)	Minimize		
Y_2_: Entrapment efficiency (%)	Maximize		

**Table 2:** Central composite design batches and their obtained responses.

	Formulation Parameters		Responses	
	X_1_: BMT conc. (mg)	X_2_: CHOL:SAA ratio	Y_1_: Vesicle size (nm)	Y_2_: Entrapment efficiency (%)
F1	1	−1	219.1 ± 9.6	75.8 ± 1.7
F2	0	0	189.9 ± 11.2	76.8 ± 1.1
F3	0	0	190.2 ± 10.3	78.3 ± 1.3
F4	−1.414	0	179.2 ± 8.5	66.2 ± 1.9
F5	−1	−1	199.1 ± 7.8	66.9 ± 1.1
F6	0	−1.414	215.6 ± 12.2	75.2 ± 0.9
F7	1	1	184.9 ± 9.7	81.8 ± 2.1
F8	−1	1	165.9 ± 6.7	74.0 ± 1.8
F9	0	1.414	170.1 ± 10.6	84.1 ± 3.2
F10	1.414	0	201.7 ± 11.6	74.8 ± 2.4
F11	0	0	188.7 ± 9.4	75.8 ± 2.2
F12	0	0	190.5 ± 11.2	78.0 ± 1.0
F13	0	0	189.7 ± 6.3	78.1 ± 1.3

Data represent mean ± SD of three independent experiments.

**Table 3:** Physicochemical properties of different BMT-ISG formulations.

Formula	Conc. of Co-Polymer (% *w*/*w*)	pH	Drug Content (%)	Sol–Gel Transition Temp. (°C)	Viscosity (cp)	
					at 25 °C	at 37 °C
F1	0	6.42 ± 0.02	98.1 ± 0.5	28.1 ± 0.5	105.9 ± 9.1	184.9 ± 11.6
F2	0.5	6.87 ± 0.03	99.4 ± 0.3	30.2 ± 0.8	116.8 ± 11.3	197.8 ± 14.5
F3	1.0	7.19 ± 0.03	99.3 ± 0.9	34.6 ± 0.9	125.3 ± 8.2	207.3 ± 10.4
F4	1.5	7.01 ± 0.04	99.1 ± 0.4	34.8 ± 0.4	144.6 ± 9.6	229.1 ± 17.3
F5	2.0	6.97 ± 0.03	97.6 ± 0.5	38.4 ± 1.8	345.1 ± 18.5	832.1 ± 35.1
F6	2.5	6.91 ± 0.02	97.3 ± 0.6	40.5 ± 1.6	402.7 ± 21.3	889.2 ± 29.7

All data represent mean ± SD of three independent experiments.

**Table 4:** Stability study of BMT-ISG formulation (F4).

Time	Visual Appearance	pH	Drug Content
0	Clear	7.01 ± 0.04	99.1 ± 0.5
4th week	Clear	6.91 ± 0.15	98.7 ± 1.1
8th week	Clear	6.75 ± 0.20	97.9 ± 1.2

Data represent mean ± SD (n = 3).

**Table 5:** Pharmacodynamic parameters following ocular administration of different BMT formulations (n = 6).

Parameter	BMT Solution	BMT-Niosomes	BMT-ISG
T_Emax_ (h)	2.00	2.00	3.00
E_max_ (mm Hg)	5.80 ± 0.6	8.6 ± 0.9	9.70 ± 0.6
AUC_E0-8h_ (mm Hg.h)	24.60 ± 3.7	53.27 ± 2.9	63.32 ± 5.8
MRT (h)	3.16 ± 0.2	8.07 ± 0.9	12.62 ± 1.3

## Data Availability

Data are contained within the article or Supplementary Materials.

## Supplementary Materials

The following supporting information can be downloaded at: https://www.mdpi.com/article/10.3390/polym15214336/s1, Figure S1: Calibration curve for UV-Visible spectrophotometric determination of BMT (λ max = 294 nm); Figure S2: FTIR spectra of pure BMT, cholesterol, Span 60, and optimized BMT-loaded niosomes; Table S1: Results of statistical analyses of all dependent variables; Table S2: Stability study results of BMT-loaded niosomes stored at 4 °C for 90 days; Table S3: Kinetic analysis of the in vitro release data of BMT from different BMT formulations.
