# Supplementary material for: Ophthalmic Bimatoprost-Loaded Niosomal In Situ Gel: Preparation, Optimization, and In Vivo Pharmacodynamics Study
Source: Polymers (Basel). 2023 Nov 6;15(21):4336. doi: 10.3390/polym15214336 (PMC10649908; doi:10.3390/polym15214336)
Supplement: Supplementary file 1 [file polymers-15-04336-s001.zip › polymers-2643878-SI.pdf]

Figure S1. Calibration curve for UV-Visible spectrophotometric determination of BMT (λ_{max} = 294 nm)

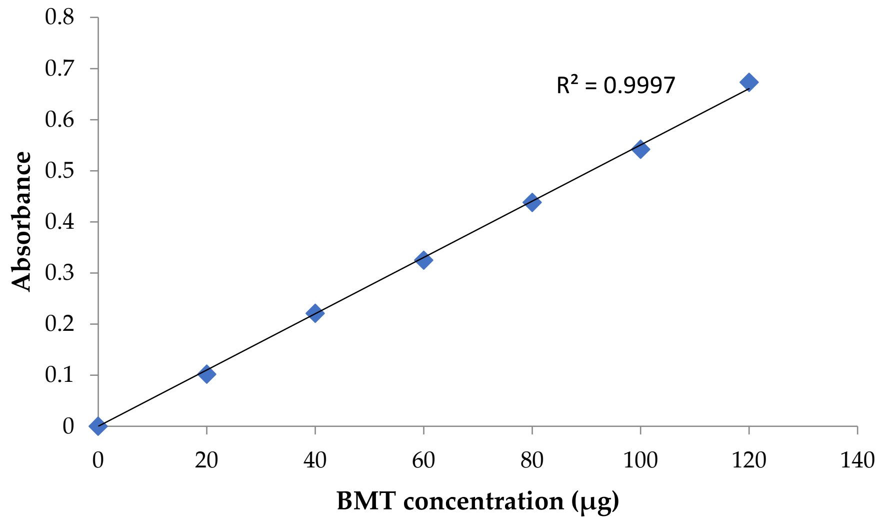

Figure S2: FTIR spectra of pure BMT, cholesterol, Span 60, and optimized BMT-loaded niosomes

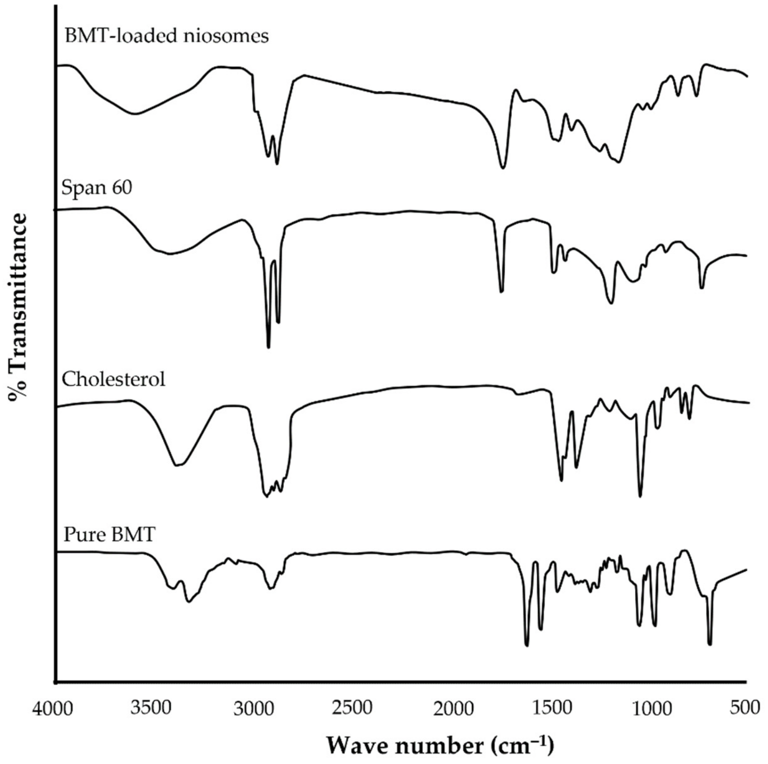

Table S1. Results of statistical analysis of all dependent variables

Source	Y_1		Y_2	
	F-Value	*p*-Value	F-Value	*p*-Value
Model	386.25	<0.0001	53.60	< 0.0001
X_1: Drug Concentration	429.84	<0.0001	96.01	<0.0001
X_2: Chol:SAA ratio	1487.56	<0.0001	76.04	<0.0001
Lack of Fit	5.91	0.0595	6.88	0.0373
R^2 analysis				
R^2	0.9964		0.9745	
Adjusted R^2	0.9938		0.9564	
Predicted R^2	0.9780		0.9042	
Adequate precision	61.72		26.56	

Table S2. Stability study results of BMT-loaded niosomes stored at 4 ˚C for 90 days

**Evaluation parameters**	**Day 0**	**Day 30**	**Day 90**
Vesicle size (nm)	167.3 ± 9.1	174.7 ± 11.1	180.2 ± 8.7
Zeta potential (mV)	– 12.4 ± 1.9	– 12.1 ± 0.9	– 11.5 ± 1.2
Entrapment efficiency (%)	81.2 ± 1.2	79.4 ± 2.4	77.3 ± 3.9

Table S3. Kinetic analysis of the *in vitro* release data of BMT from different BMT formulations

**Formula**	**Zero-order**	**First-order**	**Higuchi-diffusion**	**Korsmeyer-Peppas**	
	r^2				**n**
Pure BMT	0.851	0.775	0.933	0.957	--
BMT-loaded niosomes	0.846	0.637	0.977	0.974	0.627
BMT-ISG	0.899	0.650	0.983	0.978	0.785
